# Deep Learning for Brain Tumor Segmentation: A Survey of State-of-the-Art

**DOI:** 10.3390/jimaging7020019

**Published:** 2021-01-29

**Authors:** Tirivangani Magadza, Serestina Viriri

**Affiliations:** School of Mathematics, Statistics and Computer Science, University of KwaZulu-Natal, Durban 4000, South Africa; 219098526@stu.ukzn.ac.za

**Keywords:** brain tumor segmentation, deep learning, magnetic resonance imaging, survey

## Abstract

Quantitative analysis of the brain tumors provides valuable information for understanding the tumor characteristics and treatment planning better. The accurate segmentation of lesions requires more than one image modalities with varying contrasts. As a result, manual segmentation, which is arguably the most accurate segmentation method, would be impractical for more extensive studies. Deep learning has recently emerged as a solution for quantitative analysis due to its record-shattering performance. However, medical image analysis has its unique challenges. This paper presents a review of state-of-the-art deep learning methods for brain tumor segmentation, clearly highlighting their building blocks and various strategies. We end with a critical discussion of open challenges in medical image analysis.

## 1. Introduction

Brain tumors are an abnormal growth of cells in the brain. Their exact causes are not yet known, but there are factors that can increase the risk of brain tumor, such as exposure to radiation and a family history of brain cancer. There has been an increase in incidences of brain tumors in all ages globally over the past few years [1]. In the United States alone, an estimate of 78,980 new cases of primary malignant and non-malignant tumors were expected to be diagonized in 2018. Despite considerable efforts in brain tumor segmentation research, patient diagnosis remains poor [2]. The most common types of tumors in adults are meningiomas (low grade tumors) and gliomas and glioblastomas (high grade tumors). Low grade tumors are less aggressive and they come with a life expectancy of several years. High grade tumors are much more aggressive and they have a median survival rate of less than two years.

Medical imaging techniques, such as Magnetic Resonance Imaging (MRI), CT scans, Positron emission tomography (PET), among others, play a crucial role in the diagnosis of the tumors. These techniques are used to locate and assess the progression of the tumor before and after treatment. MRI is usually the modality of choice for diagnosis and treatment planning for brain tumors [2] due to its high resolution, soft tissue contrast, and non-invasive characteristics. Surgery is the most common form of treatment for brain tumors, but radiation and chemotherapy can also be used to slow the growth of the tumor [1]. More than one MRI slice is required to view different regions of the brain, e.g., T1, T2, T1 contrast and FLAIR images.

Again, in clinical practice, delineation of the tumor is usually done manually. An experienced radiologist will carefully study the scanned medical images of the patient segmenting all of the affected regions. Apart from being time consuming, manual segmentation is dependent on the radiologist and it is subject to large intra and inter rater variability [3]. Consequently, manual segmentation is limited to qualitative assessment or visual inspection only.

Meanwhile, quantitative assessment of the brain tumors provides valuable information for a better understanding of the tumor characteristics and treatment planning [4]. Quantitative analysis of the affected cells reveals clues about the disease progression, its characteristics, and effects on the particular anatomical structure [5]. This task proved to be difficult, because of large variability in shape, size, and location of lesions. Moreover, more than one image modalities with varying contrast need to be considered for accurate segmentation of lesions [4]. As a result, manual segmentation, which provides arguably the most accurate segmentation results, would be impractical for larger studies. Most research endeavors today now focus on using computer algorithms for the automatic segmentation of tumors with the potential to offer objective, reproducible, and scalable approaches to the quantitative assessment of brain tumors.

These methods categorically fall into traditional machine learning and deep learning methods [6]. The application of statistical learning approaches to low-level brain tumor classification features is common in conventional machine learning methods. They mainly focus on the estimation of tumor boundaries and their localization. Additionally, they heavily depend on preprocessing techniques for contrast enhancement, image sharpening, and edge detection/refining, relying on human expertise for feature engineering. Wadhwa et al. [7] provide a concise overview of methods in this category.

On the other hand, deep learning methods rely on large scale dataset availability for training and require minimum preprocessing steps than traditional methods. Over the past few years, convolutional neural networks (CNNs) have dominated the field of brain tumor segmentation [6]. Alom et al. [8] provide a detailed review of deep learning approaches that span across many application domains.

Preliminary investigations [9,10] saw deep learning as a promising technique for automatic brain tumor segmentation. With deep learning, a hierarchy of increasingly complex features is directly learned from in-domain data [1] bypassing the need of feature engineering as with other automatic segmentation techniques. Accordingly, the focus would be on designing network architectures and fine-turning them for task at hand. Deep learning techniques have been popularized by their ground breaking performance in computer vision tasks. Their success can be attributed to advances in high-tech central processing units (CPU) and graphics processing units (GPUs), the availability of huge datasets, and developments in learning algorithms [11]. However, in the medical field, there is hardly enough training samples to train deep models without suffering from over-fitting. Furthermore, ground truth annotation of three-dimensional (3D) MRI is a time consuming and a specialized task that has to be done by experts (typically neurologists). As such, publicly available image datasets are rare and will often have few subjects [12].

In this survey, we highlight state of the art deep learning techniques, as they apply to MRI brain tumor segmentation. Unique challenges and their possible solutions to medical image analysis are also discussed.

## 2. Overview of Brain Tumor Segmentation

This section provides a brief introduction to brain tumor segmentation.

### 2.1. Image Segmentation

A digital image, like an MRI image, can be represented as a two-dimensional function, f(x,y), where *x* and *y* are the spatial coordinates and the value of *f* at any given point (x,y) is the intensity or gray level of the image at that point. Each point in an image represents a picture element, called a pixel. The function *f* can also be viewed as M×N matrix, *A*, where *M* and *N* represent the number of rows and columns, respectively. Thus,

(1) $$A=f\left(x,y\right)=\left[\begin{array}{ccc} a_{1,1} & a_{1,2} & \cdots \\ \vdots & \ddots & \\ a_{M,1} & \; & a_{M,N} \end{array}\right]$$

In computer vision, image segmentation is the process of partitioning a digital image into multiple disjoint segments, each having certain properties. It is typically used in order to locate objects and their boundaries in images. This is achieved by assigning every pixel. (x,y), in an image *A*, a label depending on some characteristics or computed property, such as color, texture, or intensity.

**Figure 1:**
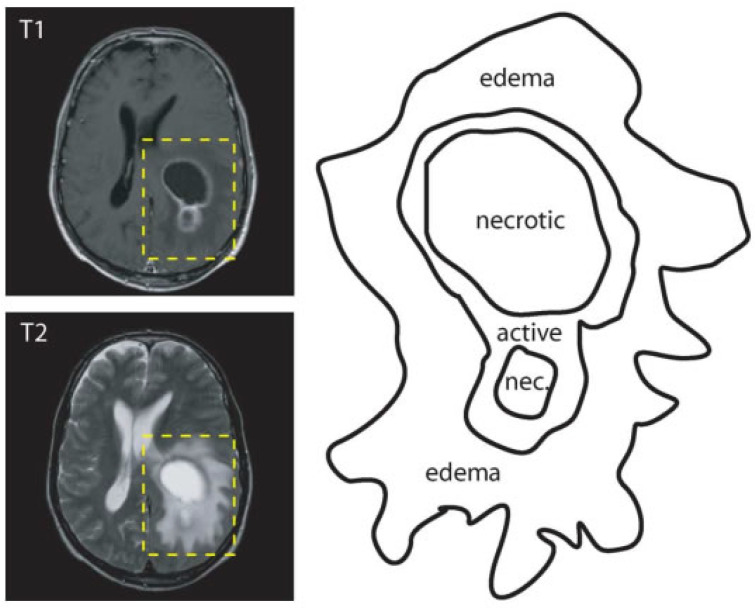
Labeled example of a brain tumor illustrating the importance of the different modalities (adapted from [13]).

The goal of brain tumor segmentation as depicted in Figure 1, is to detect the location, and extension of the tumor regions, namely:

- active tumorous tissue;
- necrotic (dead) tissue; and,
- edema (swelling near the tumor).

This is done by identifying abnormal areas when compared to normal tissues [1]. Some tumors, like glioblastomas, are hard to distinguish from normal tissues, because they infiltrate surrounding tissues causing unclear boundaries. As a solution, more than one image modalities with varying contrasts are often employed. In Figure 1, two MRI modalities (T1 with contrast and T2) were used in order to accurately delineate tumor regions.

### 2.2. Types of Segmentation

Brain tumor segmentation can be broadly categorised as manual segmentation, semi-automatic segmentation, and fully automatic segmentation, depending on the level of human involvement. Gordillo et al. [14] provide a full description of these methods.

#### 2.2.1. Manual Segmentation

With manual segmentation, a human operator uses specialized tools in order to carefully draw or paint around tumor regions. The accuracy of segmentation results depends heavily on the training and experience of the human operator as well as knowledge of brain anatomy. Apart from being tedious and time consuming, manual segmentation is widely used as a gold standard for semi-automatic and fully automatic segmentation.

#### 2.2.2. Semi-Automatic Segmentation

Semi-automated segmentation combines both computer and human expertise. User interaction is needed for the initialisation of the segmentation process, providing feedback and an evaluation of segmentation results [3]. Although semi-automatic segmentation methods are less time consuming than manual segmentation, their results are still dependent on the operator.

#### 2.2.3. Fully Automatic Segmentation

In fully automatic brain tumor segmentation, no human interaction is required. Artificial intelligence and prior knowledge are combined in order to solve the segmentation problems [3]. Fully automatic segmentation methods are further divided into discriminating and generative methods. Discriminating methods often rely on supervised learning where relationships between input image and manually annotated data are learnt from a huge dataset. Within this group, classical machine learning algorithms, which rely on hand crafted features, have been extensively used with great success over the past years. However, these methods may not be able to take full advantage of the training data due to the complexity of medical images [15]. More recently, deep learning methods have gained popularity because of their unprecedented performance in computer vision tasks and their ability to learn features directly from data. On the other hand, generative methods use prior knowledge regarding the appearance and distribution of difference tissue types.

## 3. Deep Learning

**Figure 2:**
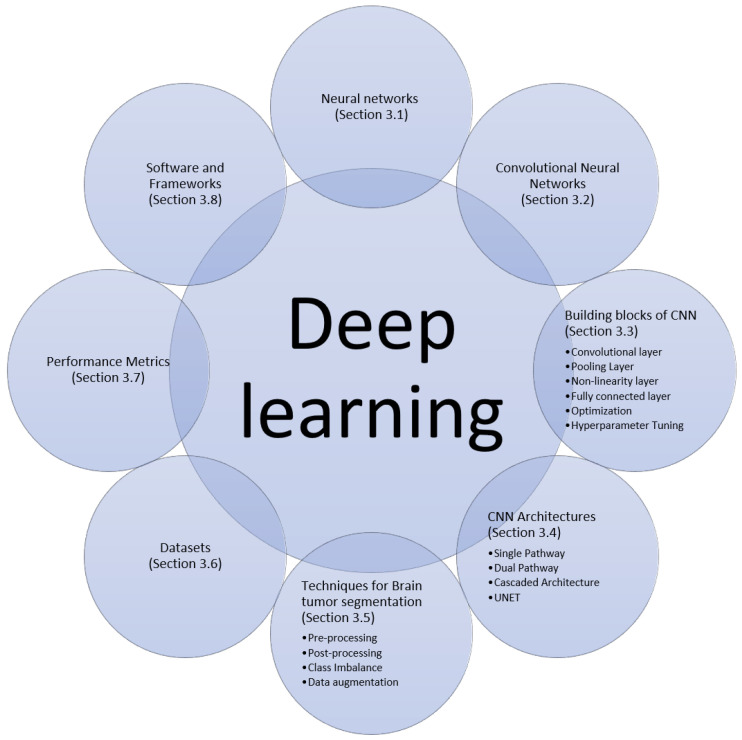
Building blocks, architectures and techniques for deep learning algorithms for brain tumor segmentation.

Deep learning is a class of machine learning algorithms that uses multiple layers to learn a hierarchy of increasingly complex presentations directly from the raw input. Machine learning models are all about finding appropriate representations for their input data. In this section, we will describe the building blocks, and recent techniques and architectures of deep learning algorithms for brain tumor segmentation that we found in papers surveyed in this work, as summarized in Figure 2.

### 3.1. Neural Networks

**Figure 3:**
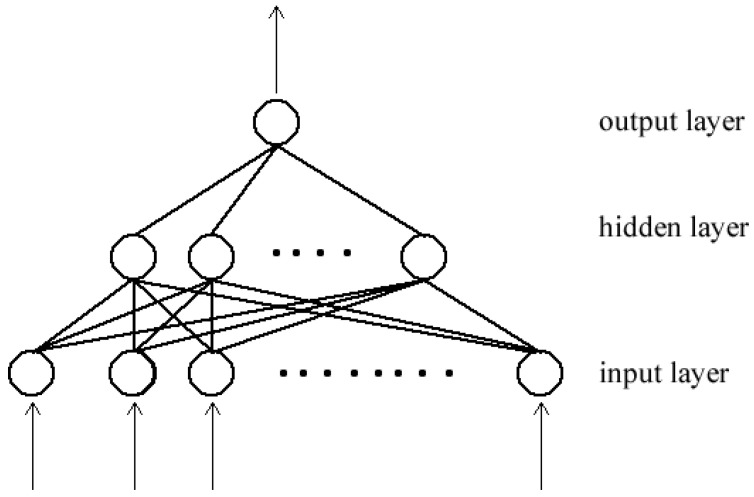
Typical feed-forward neural network composed of three layers. (adapted from [18]).

A neural network is a type of a machine learning algorithm that is able to learn useful representations from data [16,17]. The network is formed by connecting processing units, called neutrons, by directed links. Each link is associated with a weight that adjusts as learning proceeds. When the topology of the network forms a directly acyclic graph, the network is referred to as a feed forward neural network (Figure 3). Associated with each neutron is a function f(x:θ), which maps an input *x* to an output *y* and it learns the value of the parameters θ={w,b}, where *w* is a weight vector and *b* is a scalar, through a back-propagation algorithm:

(2) f(x:θ)=σ(w·x+b)

where σ(·) is element-wise non-linearity activation function.

In a typical neural network, neurons are organized in layers. The input of each neuron in a layer is connected to all or some of the output of neurons in the up-stream layer. Likewise, the output of each neuron is connected to all or some of the input of neurons in the downstream layer. The first layer in the network is the input layer, and the final layer is the output layer. Layers in the middle are referred to as hidden layers. When each neuron in a layer is connected to all of the neurons in the next layer, the network is called fully connected network. A deep neural network is formed when there are many hidden layers, hence the term *deep learning*.

### 3.2. Convolutional Neural Network (CNN)

A convolutional neural network is a type of a neural network that performs a convolutional operation in some of its layers. The convolutional layer is able to learn local features from the input data. By stacking many convolutional layers one after the other, the network is able to learn a hierarchy of increasingly complex features. A polling layer is usually added in-between successive convolutional layers to summarize important features. This will reduce the number parameters that are passed to downstream layers and, at the same time, introducing translation invariant (able to recognize learned patterns, regardless of their geometric transformations) to the network.

Recently, CNN has become the de factor model for brain tumor segmentation because of its record shattering performance in classical computer vision problems as well as in medical image analysis as compared to other models. CNN models are able to learn spatial hierarchies of features within data, for example, the first convolutional layer will learn small local patterns, like edges, the second layer will learn larger patterns made up of features of the preceding layer and so on. This ability make them a better fit for image analysis task. Furthermore, units in convolutional layers share weights, thereby reducing the number of parameter to learn and improve the efficiency of the network.

### 3.3. Building Blocks CNN

#### 3.3.1. Convolutional Layer

This layer consists of a set of learnable filters or kernels (the typical size is usually 3 × 3 or 3 × 3 × 3, depending whether the input is a two-dimensional (2D) or three-dimensional (3D) image, respectively) that are used to slide over the entire input volume, performing a dot product between entries of the filter and the input at that point. Thus, the convolutional operation first extracts patches from its input in a sliding window fashion, and then applies the same linear transformation to all of these patches. The output of the convolution operation is sometimes referred to as the feature map. The network will learn filters that recognize certain visual patterns present in the input data. When convolutional layers are stacked one after the other, the network is able to learn a hierarchy of increasing complex features, from simple edges to being able to recognize the presence of a face for example.

Over the past few years, there were various attempts meant to improve the performance of deep learning models by replacing the conventional convolutional layer with blocks that increase the network’s capacity while using less computational resources. For example, Szegedy et al. [19] introduced the inception block that captured sparse correlation patterns whlie using multi-scale receptive fields. Their network architecture, the GoogleNet, a winner of ILSVRC 2014, had fewer network parameters and required less computational resources than its predecessors AlexNet [20] or VGG [21]. The residual block was another notable improvement [22], which facilitated very deep networks that do not suffer from the vanishing gradient problem. Hu et al. [23] introduced the Squeeze-and-Excitation (SE) block that captured the interdependencies between the network’s feature maps.

#### 3.3.2. Pooling Layer

A pooling layer usually follow a convolutional layer or a set of convolutional layers. The goal is to reduce the dimensions of the feature maps, and at the same time, keep important features. A pooling operation is applied to a rectangular neighbourhood in a sliding window fashion. For example, the max pooling is used in order to produce a maximum of a rectangular neighbourhood. Other popular pooling operations include average and weighted average pooling.

#### 3.3.3. Non-Linearity Layer

Typical convolutional layers involves three steps [16]. In the first step, the layer performs convolutional operation on input feature maps to produce a set of linear activations. Second, a non-linear transformation is performed on the output feature maps. Third, a pooling layer is used in order to modify the output further. Non-linear transformations can be obtained by using special class of functions, called activation functions. Non-linearity gives the network the ability to learn nontrivial representations that are sparse. Hence, making the network resilient to slight modifications or noise in the input data as well as improving computational efficiency of the representations.

In the past, sigmoid and hyperbolic tangent functions were commonly used for the non-linearity layer. Today, the most popular activation function is the rectified linear unit (ReLU), which is expressed as f(z)=max(z,0). It was observed in [20,24], where ReLU typically learns faster in network with many layers and does not suffer from vanishing/exploding gradients, as with the sigmoidal activations. However, ReLU presents some potential drawbacks when the network saturates with a constant zero gradient causing the network to converge slowly. As a solution, Maas et al. [25] proposed a Leaky ReLU (LReLU) that allows for small, non-zero gradient to flow when the network is saturated. This function is defined as

(3) $$\begin{array}{c} f(z)=max(z,0)+\alpha min(0,z) \end{array}$$

where α is a constant leakiness parameter(typically 0.01). Another common variant of ReLU is Parametric Rectified Linear Unit (PReLU) [26]. This activation function adaptively learns the parameter α in Equation (3), thus improving the accuracy with less computational cost.

#### 3.3.4. Fully Connected Layer

The convolutional layers are used as feature extractors. The features that they produce are then passed to the fully connected (FC) layers for classification. Each unit in the FC layer is connected to all of the units in the previous layer, as shown in Figure 3. The final layer is usually a softmax classifier, which produces a probability vector map over the different classes. All of the features are converted in to a one-dimensional feature vector before being passed to a FC layer. By doing so, spatial information inherent in image data is lost. Another issue with the FC layers is that they have a larger number of parameters as compared to other layers that increase the computational costs and require input images to be of the same size.

As a solution to above problems, Long et al. [27] proposed converting FC layers to 1 × 1 convolutional layers, thus transforming the the network into a fully convolutional network (FCN). The network takes the input of any arbitrary sizes and outputs a grid of classification maps.

#### 3.3.5. Optimization

The performance of the deep CNN can be improved (or optimized) by training the network on a large dataset. Training involves finding the parameters θ of the model that significantly reduce a cost function J(θ). Gradient descent is the widely used method for updating network parameters through a back-propagation algorithm. Optimization can be done per single sample, subset, or full set of the training samples. Thus, stochastic, mini-batch, or batch gradient descent, respectively. Today, many optimization algorithms for deep learning use mini-batches and it is now common to just call them stochastic methods [16].

Stochastic gradient descent (SDG) comes with few notable challenges. Choosing an appropriate learning rate can be difficult. A learning rate that is too small leads to very slow convergence (tiny updates to the model parameters) and, at the same time, too large will result in undesired divergence behavior in the loss function. All of the parameter updates are based on the same learning rate, disregarding the fact that some of the features might have higher frequency than other. Another key challenge is that optimization can be trapped in sub-optimal local minima or saddle points, especially for non-convex optimization [28].

Various variants of SDG have been proposed in the literature that address the aforemented challenges. Memontum-based SDG methods [29] can help in accelerating SDG in relevant direction, dampening undesirable oscillations in local optima. Adagrad [30] addressed the issue of manually turning the learning by adapting the learning rate to the parameters, performing larger updates for infrequent parameters as compared to frequent ones. However, Adagrad suffers from monotonically decreasing learning rate to a point at which the algorithm stops learning. Adadelta [31], RMSprop [32], and Adam [33] addressed the shortcomings of Adagrad by dividing the learning rate by an exponentially decaying average of past gradients.

#### 3.3.6. Loss Function

In machine learning, a loss function is used in order to evaluate how well a specific algorithm models the given data. When the output is far from the true value, loss will be very high and low when the predictions are close to the true values. The primary goal of training a neural network is to minimize the loss (or cost) function of the network as much as possible and, at the same time, ensuring that the network generalizes well with unseen data.

The choice of the cost function depends on the problem area, whether it is a classification or regression problem and the choice of the output unit [16]. The majority of the image classification algorithms use softmax loss, withhs a combination of softmax and CE loss or log-loss [28]. The softmax function produces a probability distribution over a number of given output classes, while the CE loss takes the probability of predictions and penalizes predictions that are confident but wrong. Class imbalance is one major issue in medical image analysis, where one class will have fewer instances than the other. For example, a brain tumor occupies a small portion when compared to healthy tissues. As a result, the classifier will tend to be biased to the majority class. One way of addressing such a problem is to adapt loss functions for class imbalance. Some works [34,35,36] proposed a loss function that is based on the Dice coefficient. Ronneberger et al. [37] proposed a weighted CE loss, which gives more importance to some pixels in the training data.

#### 3.3.7. Parameter Initialization

Deep learning optimization algorithms are iterative in nature, thus requiring the user to specify initial starting point of the algorithms [16]. The choice of initialization will influence how quickly learning can converge if it can converge at all. Empirical studies have shown that a carefully chosen initialization scheme dramatically improves the rate of convergence [38], while gradient-based optimization starting from random initialization may get stuck near poor solutions [39].

Ref. [38] proposed a normalized initialization scheme (Xavier initialization), which guarantees that weight initialization should not obtain values that are too small or too large, thus reducing saturation and vanishing gradients, thereby improving convergence. This approach was later improved in [26] to perform much better on Relu or PRelu activations and extreme deep models.

#### 3.3.8. Hyperparameter Tuning

Hyperparameters are parameters that are supplied by the user to control the algorithm’s behavior before training commences, such as learning rate, batch size, image size, number of epochs, kernel size etc. While the learning algorithms do not adapt these parameters, their choice has varying effects on the resulting model and its performance. The majority of the works studied in this review set their hyperparameters manually or perform a grid search while using the validation set. However, these approaches will become impractical when the number of hyperparameters is large [40] and they rely on human expertise, intuition, or guessing. As a solution to these challenges, automated approaches, like AutoML (http://www.automl.org) and Keras Tuner, (https://keras-team.github.io/keras-tuner/) are beginning to gain much attention.

#### 3.3.9. Regularization

Regularization is a technique for improving the performance of a machine learning algorithm on unseen data. It is a way of reducing over-fitting on training set. Over-fitting occurs when the gap between the training error and test error is too large [16]. When that happens, the model performs well on training data, but poorly on previously unseen data. There are various techniques that can be employed in order to reduce the generalization error, such as reducing the model capacity, which is, reducing the number of learnable parameters in the model; adding $L^{2}$ or $L^{1}$ weight decay regularization term to the cost function to force the model to only take small weight values; introducing early stopping whenever the model performance stops improving on validation dataset; randomly dropping out (skipping) the output of some units during training [41]. The last approach is one of the most effective and most commonly used technique [17], mainly because it is computationally inexpensive and prevents interdependent learning amongst units. Batch Normalization [42] can also be used as a regularizer by ensuring that the distribution of non-linearity inputs remains more stable as the model trains, thereby improving the training of the model.

Training a machine learning model with more data is the best way to reduce the generalization error. However, in the medical domain, acquiring a training dataset is time-consuming, more expensive, and requires highly trained personnel to annotate ground truth labels. Data augmentation can increase the dataset and reduce over-fitting by flipping, applying small rotations, warping, and using the non-rigid deformation transformation of images. However, great care must be taken when performing transformations of the medical image dataset since the patch’s label is determined by the center of pixel [43]. Some recent works used generative models that include variational autoencoders [44] and generative adversarial networks [45] to act as additional regularization that deals with data scarcity.

### 3.4. Deep CNN Architectures

#### 3.4.1. Single Pathway

A single pathway architecture is a basic network that resembles a feed-forward deep neural network. Data flows from the input layer to the classification layer using a single path. Urban et al. [10] proposed a 3D single path CNN which has fully connected convolutional layer as the classification layer. This gave the network the ability to classify multiple 3D pixel in one go. In [46], each image’s modality was fed to a different two-dimensional (2D) CNN. The result of each CNN was then used as features to train a random forest classier. Extracts from XY, XZ, and YZ planes around each center pixel were used as the neighborhood information. Pereira et al. [43] used small kernels in their convolutional layers. As a result, a very deep network, DeepMedic, was obtained, which can learn more feature hierarchies. Their architecture obtained first and second positions in BRATS 2013 and 2015 challenge, respectively.

#### 3.4.2. Dual Pathway

Many segmentation algorithms perform pixel-wise classification, where an input patch is extracted from an MRI image and then predicts the label of the central pixel without considering global neighborhood information. This can be risky because of infiltrating nature of brain tumors, which produces unclear boundaries. Hence, local information cannot be enough to accurately produce good segmentation results. As a solution, other researchers [1,47] introduced neighbourhood information to the mix by using CNN with two data streams (dual pathway) that are combined in order to influence label predictions of each pixel. One of the streams will represent local information, the visual details of the region around the center pixel. The other stream represents the global context, which takes the location of the extracted patch in the brain into account.

#### 3.4.3. Cascaded Architecture

In a cascaded architecture, the output one CNN is concatenated with the other. There many variations with this architecture in the literature, but the most prominent is the input cascade [1,48]. In this architecture the output of one CNN becomes a direct input of another CNN. The Input cascade is used in order to concatenate contextual information to the second CNN as additional image channels. This is an improvement to the dual-path way that performs multi-scale label predictions separately from each other. Another variation of cascaded architecture is the local pathway concatenation [1]. In this architecture, the output of the first CNN is concatenated with the output of the first hidden layer of the second CNN instead of its input.

Hierarchical segmentation [34,49] is another form of a cascaded architecture. In this architecture, the segmentation of brain tumor regions is sequentially done by reducing the multi-class segmentation problem into the multi-stage binary segmentation problem. This architecture takes full advantage of the hierarchical nature of tumor sub-regions and helps in reducing false positives as well as mitigating the inherent class imbalance problem. The first stage of architecture segments the whole tumor from the input MRI modalities, which is then used as a bounding box for the next stage. For the second stage, the output of the first stage is used as an input to perform either a multi-class intra-tumoral segmentation, as in [49], or perform successive binary segmentation of the remain tumor sub-regions [34]. Wang et al. [34] observed an increase in the training and inference time of a multi-stage binary segmentation as compared to a single multi-class network approach.

#### 3.4.4. UNET

The UNET architecture [37] is an improvement of FCN [27], which resembles an encoder and decoder network designed specifically for biomedical image segmentation. The network consists of a contracting path (encoder) and an expansive path (decoder), which gives it the u-shaped architecture. The contracting path consists of the repeated application of two convolutional layers, followed by a rectified linear unit (ReLU) and max pooling layer. Along the path, the spacial information is reduced, while feature information is increased. The expansive path consists of a series of up-sampling operations combined with high-resolution features from the contracting path through skip connections.

### 3.5. Techniques for Brain Tumor Segmentation

#### 3.5.1. Pre-Processing

Data preprocessing is a very crucial step of preparing raw input data to be more amenable to neural networks. MRI images contains various artifacts that are caused by the acquisition protocol and the hardware used. These artifacts need to be corrected before the images are fed into the network for better performance. One of the notable artifacts is the presence of smooth intensity variations within the image, which is also known as bias field. Among various techniques for bias field correction, the non-parametric nonuniform normalization (N3) [50] approach has become the technique of choice for bias field correction due to its ease of use and its availability as an open source project [51]. This technique was later improved in [51] and it is also well known as N4ITK. These techniques are limited to a single image. Accordingly, for uniform intensity distribution across patients and acquisitions, the intensity normalization proposed by Nyul et al. [52] can be applied.

Another popular preprocessing technique is to normalize image dataset to have a mean zero and a standard deviation of one. This technique assists in removing the bias from features. Image cropping can also be applied to remove as much background pixels as possible.

#### 3.5.2. Post-Processing

The post-processing step is performed to further refine the segmentation results. It helps in reducing the number of misclassifications or false positives in the segmentation results while using algorithms, like conditional random fields (CRF) [4,34,53], markov random fields (MRF) [54], connected component analysis [1,53,55], and morphological operators [48,56]. CRF and MRF based techniques effectively remove false positives by combining model predictions with low-level image information, like local interations of pixels and edges when making finer adjustments. However, these techniques are computationaly expensive [14]. Connected compents analysis involves finding and extracting connected components and then applying a simple thresholding technique to remove unwanted blobs. Another technique of removing false positive around edges of the segmentation image is to apply morphological operations, erosion, and dilation in succession.

#### 3.5.3. Class Imbalance

The performance of the segmentation task is affected by the class imbalance problem, where there is an unequal distribution of voxel classes in the training dataset. For example, in brain tumor segmentation, healthy voxels constitute 98% of the total voxels [1]. Training the model on this distribution will cause the model to be more biased towards the majority class. Whereas, training with equal distribution results in bias towards tumor classes [57]. Several techniques have been explored in the literature in order to address this problem.

Many works incorporated loss-based methods of addressing the class-imbalance problem. Lin et al. [58] proposed a loss function that addresses the problem by dynamically scaling the loss based on the model’s confidence in classifying samples. The scaling factor was reduced when the model’s accuracy in classifying classed increases. As a result, the model pays more attention to misclassified samples. In [59], dice loss was used as a means of addressing the problem. Some works [60,61] incorporated a weighted-loss function, where voxels (or pixels) belonging to different classes are assigned weights according to their distribution in the training data. This ensures that each class in the segmentation problem has an equal contribution to the model’s loss. Kuzima et al. [62] combined the CE loss with Dice based loss as means of addressing class imbalance problem. Other works explored hard negative mining [63,64] as a solution to the class-imbalance problem. Voxels with largest negative losses and positive voxels are used in order to update the model’s weights.

Two-phase training [1,5,57] is also another way of dealing with the class imbalance problem. In the first phase, the network is trained with patches that have equal class distribution and then trained with true class distribution in the second phase. Hussain et al. [57] reported that two-phased training helped in removing most of the false positives.

In [34], Wang et al. pointed out that hierarchical segmentation also assists in addressing the class-imbalance problem.

#### 3.5.4. Data Augmentation

Data augmentation is a technique for reducing the generalization error of a machine learning algorithm. As indicated earlier, one way of effectively increasing the machine learning model’s generalization capabilities is to train it on more data. However, acquiring a considerable amount of high-quality training data is nearly impossible in practice, especially for the medical domain. Data augmentation has emerged in order to increase the training data by creating more synthetic data and adding (augment) it to the training set.

Data augmentation can be broadly divided into two categories [65]: the transformation of original data and artificial data generation. With the transformation of original data, new data are generated by applying various transformations on the original data, which include affine transformations (which involves rotation, zooming, cropping, flipping, and translations), elastic transformations (shape variations), and pixel-level transformation (intensity variations). While these transformations assist in mitigating insufficient data challenges, they fundamentally produce very correlated images [66], which results in very little performance improvement [66,67] and sometimes generates anatomically incorrect examples (e.g., using rotation) [65]. However, their use in the literature is widespread, due to the ease of implementation.

On the other hand, artificial data generation [67,68] exploits the Generative adversarial networks (GANs) [69] to generate realistic data that are indistinguishable from the real data and also serves as a effective method for data anonymization [66]. GANs are able to generate a wide variety of realistic samples that can bring invariance and robustness. However, there are scenarios where they can generate samples that are very similar to the real ones, resulting in poor performance [65].

### 3.6. Datasets

Over the past few years, there have been considerable research interests in automatic brain tumor segmentation. As research output continued to grow, the objective evaluation of different algorithms became a challenge because researchers used private datasets with varying attributes. As a result, benchmarking challenges, such as Multi-modal Brain Tumor Image Segmentation (BRATS), emerged to standardize performance evaluation while using publicly accessible datasets. Table 1 show a summary of the mostly used datasets for brain tumor segmentation.

Since 2012, the BRATS Challenge [2], in conjunction with the International Conference on Medical Image Computing and Computer-Assisted Interventions (MICCAI), has been the primary bench-marking resource for brain tumor segmentation. It offers the medical research community publicly accessible datasets for training and validation and standardized metrics in order to objectively evaluate model performance against an online evaluation platform. The dataset initially contained as small as 30 clinically acquired scans of glioma patience, and the number has continued to grow over the subsequent years.

Medical Segmentation Decathlon Challenge offers a relatively large dataset that supports a wide range of segmentation task. The Challenge aims to facilitate research in general-purpose segmentation algorithms that solve various functions without any human intervention. For brain tumor segmentation, the dataset comprises a subset of the 2016 and 2017 BRATS Challenge data.

### 3.7. Performance Evaluation Metrics

In order to objectively measure the performance of segmentation algorithms, researchers have to group different tumor structures into three mutually inclusive regions:

- the *whole* tumor (includes all tumor structures);
- the *tumor* core (exclusive of edema); and,
- the *active* tumor (only consists of the "enhancing core").

Subsequently, they measure the algorithm’s performance on each region against several metrics that include the Dice score, Sensitivity, Specificity, and Hausdorff measure.

### 3.8. Software and Frameworks

Researchers and engineers have always relied on open-source software frameworks from idea generation to experimentation to production deployments in order to accelerate the deep learning workflow. This section described some of the popular machine learning frameworks that were used in the reviewed papers.

Theano [71] is a free and open-source python framework for the fast computation of large-scale dataflow mathematical expressions compiled and executed naively on both CPUs and GPUs. Moreover, the research community has been utilizing the platform in order to conduct machine learning research. However, it is not a purely a machine learning framework, but rather a compiler for mathematical expressions that are defined in NumPy-like syntax. Several high-level software packages like Pylearn2, Keras, blocks, and Lasagne have been built on top of Theano, leveraging its strengths as an efficient mathematical powerhouse.

Pylearn2 [72] is a free and open-source machine learning library that is built on top of the Theano framework. It started gaining popularity after being used to win a transfer learning challenge and implementing various state of the art computer vision benchmarks. The library focuses on flexibility and extensibility, allowing for researchers to implement arbitrary machine learning models at ease. Unfortunately, the library no longer has an active developer and has, ever since, fallen behind other actively maintained frameworks, like Keras.

Caffe [73] is a C++ deep learning framework that was initially developed for computer vision applications and later spread to other domains like robotics, neuroscience, and astronomy. It offers a complete toolkit for a deep learning pipeline, from training to production deployment. Each processing stage is supplemented with well-documented examples. Moreover, the framework is shipped with implementations of popular deep learning building block and reference models allowing for quick experimentation with state-of-the-art deep learning methods. The definition of models is done in config files, rather than being hard-coded, ensuring the separation of representation from implementation.

Pytorch [74] is yet another fully-fledged open-source deep learning framework. Its design philosophy moved away from the define and execute style, as in many frameworks that create a static computational graph before running the model. While this approach is powerful, it sacrifices usability, the ease of debugging, and flexibility. Instead, Pytorch took an imperative approach by dynamically constructing the computational graph, allowing for the models to be idiomatically defined following the python programming model. The framework also offers a seamless transition from research to production, distributed training, and the seamless execution of models on edge devices.

Tensorflow [75] is an end-to-end distributed deep learning platform for large scale machine learning applications. The platform supports the execution of dataflow graphs across a span of heterogeneous devices, such as mobile devices and large-scale distributed systems, with little or no change. Its design philosophy has been used to simplify model parallelism within a single machine and across thousands of distributed systems. It has a complete toolbox for quick experimentation with state-of-the-art deep learning models, seamless transition from research to heterogeneous deployments, and the visualization and debugging of large-scale models.

Keras [76] is a fast-growing high-level API for deep learning applications. Although it initially supported multiple data-flow graph back-ends, like Theano, it is now deeply woven into the Tensorflow 2 ecosystem. It provides consistent and simple APIs to quickly experiment with new models and leverage Tensorflow in order to export the models to run in browsers and mobile devices. Moreover, it comes bundled with building blocks and pre-trained state-of-the-art models for various machine learning domains. The industry and the research community have adopted the platform, because of its ease of use, user-centric approach, and extensive documentation.

## 4. Discussion

Deep learning methods to medical image analysis have received tremendous attention over the past few years. This is evident in the considerable increase in the number of published works each year [2]. Deep learning techniques are able to learn a hierarchy of increasingly complex features directly from data, as stated earlier. For example, in brain tumor segmentation, deep learning algorithms can learn to segment MRI images by being trained on a sufficiently large dataset. For this reason, CNN based models have been widely adopted in medical image analysis, following their success in solving many problems in computer vision, speech recognition, and natural language processing. Table 2 shows a summary of deep learning methods that were reviewed in this work. Many techniques differ considerably in terms of architectural design, with recent works following the Unet [37] architecture and ensemble methods as shown in Table 3. Moreover, several techniques have been developed in order to address inherent problems in automated brain MRI analysis.

Deep learning algorithms require a relatively large amount of training data to generalize well on unseen data. However, this poses many challenge in the medical domain. Firstly, it takes a well trained radiologist a considerable amount of time to annotate even a single MRI volume. Moreover, the work is subject to an intra-rater and inter-rater variability. Therefore, all of the annotations are approved by one to many experienced neuro-radiologists [105], before they can be used in supervised training, which makes the process of creating training and testing datasets not only time consuming, but expensive. Secondly, medical data is protected by data protection laws that restrict the usage and sharing of this kind of data to other parties. Consequently, a lot of time is spent seeking approvals and removing personal identifiable information from medical data. Fortunately, Table 1 shows a consistent increase of training and testing data for the BraTS Challenge. Hopefully, this trend will continue in the coming years. Thus, facilitating training relative deep networks and reducing over-fitting.

Because the lack of large-scale datasets restricts deep learning models’ full potential, researchers have adopted data augmentation as an immediate solution to the data challenges that are mentioned above. Other works have recently explored weakly-supervised learning [106,107,108] as a promising solution to address the need for fully annotated pixel-wise labels. Instead of performing pixel-level annotations, known to be tedious and time-consuming, weakly-supervised annotation uses bounding box or image-level annotations in order to signify the presence or absence of lesions in images. This approach has the benefit of being cheap, contains less labeling noise [107], far larger volumes of data can be generated than pixel-level annotation, and training of deep learning models can leverage both kinds of datasets.

Moreover, deep learning techniques require a huge amount of computational and memory resources [28]. Very deep networks, which are becoming a widespread, have millions of parameters that result in many costly mathematical computations that are restrictive on the kind of computational hardware that can be used by researchers. Furthermore, the use of 3D deep learning models increases the computational and memory requirements by large margins. All of the reviewed literature use deep learning software libraries to provide an infrastructure to define and train deep neural networks in parallel or distributed manner while leveraging multi-core or multi-GPU environments. Currently, researchers are being limited by the amount of GPU memory at their disposal (typically 12 gigabytes). For this reason, batch sizes and model complexities are being limited to what can fit into the available memory.

The performance of brain tumor segmentation algorithms have continued to increase over the past few years due to the availability of more training data and use of more sophisticated CNN architectures and training schemes. However, their robustness is still lagging behind expert performance [105]. Recently, researchers have used the ensemble methods to achieve state-of-the-art performance (see Table 3). Precisely, the ensemble methods fuse the segmentation results of several models to improve the robustness of individual approach, resulting in superior performance as compared to inter-rater agreements [105]. Interestingly, single Unet [37] based models [91] continue to produce exceptional performance, supporting the argument that: “*a well trained Unet is hard to beat*” [88]. The reviewed literature have shown that careful initialization of hyper-parameters, a selection of pre-processing techniques, employing advanced training schemes, as well as dealing with the class imbalance problem will immensely improve the accuracy and robustness of segmentation algorithms.

## 5. Summary

This paper has discussed several building blocks, state-of-the-art techniques, and tools for implementing automatic brain tumor segmentation algorithms. Despite the tremendous advance in the field, the robustness of deep learning methods are still inferior to expert performance. Some notable architectures, including ensemble methods and UNet based models, have shown great potential for improving the state-of-the-art with careful pre-processing, weight initialization, advanced training schemes, and techniques in order to address inherent class imbalance problems. The lack of a large-scale medical training dataset is the leading factor in many segmentation algorithms’ poor performance.

## Figures and Tables

**Table 1:** Summary of commonly used public datasets for brain tumor segmentation.

Name	Total	Training Data	Validation Data	Testing Data
BRATS 2012 [2]	50	35	-	15
BRATS 2013 [2]	60	35	-	25
BRATS 2014 [2]	238	200	-	38
BRATS 2015 [2]	253	200	-	53
BRATS 2016 [2]	391	200	-	191
BRATS 2017 [2]	477	285	46	146
BRATS 2018 [2]	542	285	66	191
BRATS 2019 [2]	653	335	127	191
Decathlon [70]	750	484	-	266

**Table 2:** Overview of Deep learning methods for brain tumor segmentation. BN = Batch normalization, GN = Group normalization, outliers = remove top 1%, hist-norms = Histogram normalization, RN = Range normalization, HS = Histogram standardization, slice-norm = Slice-based normalization, PLN = Piece-wise linear normalization, IN = Instant normalization, CE = Cross entropy, BS = Bootstrapping, SS = Sensitivity-specification, NM = Negative Mining, WCE = Weighted cross-entropy, neg-mining = Hard negative mining.

Reference	Input	Preprocessing	Regulization	Loss	Optimizer	Activation
*Unet Architecture*						
[47]	3D	Z-score				ReLu
[77]	2D		BN	Dice, WCE,	Adam	ReLU
				BS, SS		
[34]	2D	Z-score, hist-norms	dropout	CE	SDG	LReLU
[78]	3D	cropping	BN	Jaccard loss, CE		PReLU
[79]		Z-score, N4ITK, lin-norm				
[80]	2D			Dice	Adam	
[81]	2D	Z-score, HM	BN	CE	Adam	ReLU
[82]	3D	bounding box	dropout	Dice	Adam	
[83]	3D	Z-score, rescaling, outliers	IN, L2	Dice	Adam	LReLU
[84]	2D	slice-norm		CE	Adam	
[85]	3D		BN	Dice	Adam	
[15]	2D	Z-score	BN	CE	Adam	ReLU
[63]	3D	Z-score	GN	CE, neg-mining	SGD	
[36]	2D	bounding-box, cropping,	BN	Dice	Adam	Relu
		Z-score, intensity-windowing				
[86]	2D	N4ITK, Nyúl	BN, spatial-dropout	CE	Adam	ReLU
[60]	2D		BN	CE		ReLU
[87]	2D	Z-score, remove outliers	BN	WCE, Dice	SGD	PReLU
[88]	3D	Z-score	IN, L2	CE, Dice	Adam	LReLU
[5]		N4ITK, remove outliers		WCE	Adam	
[35]	2D	Z-score	BN	Dice	Adam	Relu
[59]	3D	Z-score	BN	Dice	Adam	PReLU
[89]	3D	Z-score	BN, L2	CE, Dice, focal	Adam	ReLU
[90]		Z-score			Adam	RelU
[91]	3D	Z-score	GN, L2, Dropout	Dice	Adam	ReLU
[92]	3D	RN, random axis mirror		CE, Dice	SDG	
[64]	3D	Z-score, N4ITK	BN, L2	CE, NM	Adam	ReLU
*Dual-pathay Architecture*						
[10]	2D		L1, L2 Dropout		SDG	
[1]	2D	Z-score, N4ITK, outliers	L1, L2, Dropout	log-loss	Maxout	ReLU
[47]	2D	Z-score			Adam	ReLU
[57]	2D	Z-score, N4ITK	BN, Dropout	log-loss	SDG	ReLU
[63]	3D		GN	CE, NM	SDG	
[53]	2D	N4ITK				PReLU
[5]		N4ITK, outliers		WCE	SGD	
[93]	3D	N4ITK, LIN				ReLU
[94]	3D		Dropout	log-loss	SDG	PReLU
[95]	2D	N4ITK	Dropout		SGD	ReLU
[4]	3D	Z-score		log-loss		ReLU
[79]		Z-score, N4ITK, PLN				
[96]	3D	Z-score	BN, L2, Dropout			ReLU
*Single-pathway Architecture*						
[9]	2D			log-loss	SGD	ReLU
[46]	2D		Dropout	CE	SGD	ReLU
[43]	2D				SGD	ReLU
[64]	3D	Z-score, N4ITK	BN	CE, NM	Adam	ReLU
[97]	2D			CE	Nesterov, RMSProp	ReLu
[98]	2D	Z-score, outliers			Adam, SGD, RMSProp	ReLu
[99]	3D					ReLU
[43]	3d	Z-score, N4ITK, Nyúl	Dropout	CE	Nesterov	LReLU
*Ensemble Architecture*						
[59]	3D	Z-score	BN	dice	Adam	PReLU
[64]	3D	Z-score, N4ITK	BN	CE, NM	Adam	ReLU
[63]	3D		GN	CE, NM	SDG	
[61]	2D	Z-score, N4ITK, HN,	Dropout	CE	Adam	
[98]	2D	Z-score, outliers			Adam, SGD, RMSProp	ReLu
[44]	3D	Z-score	GN, L2, spatial dropout	Dice	Adam	ReLU
[79]		Z-score, N4ITK, PLN				
*Cascaded Architecture*						
[34]	2D	HS, Z-score	dropout	CE	SGD	LReLU
[1]	2D	Z-score, N4ITK, remove outliers	Dropout L2, L1	log-loss	Maxout	
[48]	2D				Maxout	RelU
[85]	3D		BN	Dice	Adam	LReLU
[100]	2D	Z-score, BN, outliers	L2, dropout	CE	SGD	ReLU
[34]	2.5D	Z-score	BN	Dice	Adam	PReLU
[59]	3D	Z-score	BN	Dice	Adam	PReLU
[89]	3D	Z-score			Adam	ReLU
[86]	2D	Z-score, N4ITK	BN, spatial dropout	CE	SDG	ReLU
[34]	3D	Z-score	BN	Dice	Adam	PReLU
[86]		N4ITK, Nyúl	BN, dropout	CE	Adam	ReLU
[35]	2D	Z-score	BN	Dice	Adam	ReLU
[91]	3D	Z-score	GN, L2, dropout	Dice	Adam	ReLU

**Table 3:** A summary of top performing methods on BraTS 2017, 2018, and 2019 validation data as reported by the online evaluation platform. ET—Enhancing tumor, WT—Whole tumor, and TC—Tumor core.

Rank	Reference	Architecture	Dice			Sensitivity			Specificity			Hausdorff 95		
			ET	WT	TC	ET	WT	TC	ET	WT	TC	ET	WT	TC
*BraTS 2017*														
1	[79]	Ensemble	0.738	0.901	0.797	0.783	0.895	0.762	0.998	0.995	0.998	4.499	4.229	6.562
2	[34]	Cascaded	0.786	0.905	0.838	0.771	0.915	0.822	0.999	0.995	0.998	3.282	3.890	6.479
3	[83]	Unet	0.776	0.903	0.819	0.803	0.902	0.786	0.998	0.996	0.999	3.163	6.767	8.642
3	[101]	SegNet	0.706	0.857	0.716	0.687	0.811	0.660	0.999	0.997	0.999	6.835	5.872	10.925
*BraTS 2018*														
1	[44]	Ensemble	0.825	0.912	0.870	0.845	0.923	0.864	0.998	0.995	0.998	3.997	4.537	6.761
2	[88]	Unet	0.809	0.913	0.863	0.831	0.919	0.844	0.998	0.995	0.999	2.413	4.268	6.518
3	[102]	Ensemble	0.792	0.901	0.847	0.829	0.911	0.836	0.998	0.994	0.998	3.603	4.063	4.988
3	[103]	Ensemble	0.814	0.909	0.865	0.813	0.914	0.868	0.998	0.995	0.997	2.716	4.172	6.545
*BraTS 2019*														
1	[91]	two-stage Unet	0.802	0.909	0.865	0.804	0.924	0.862	0.998	0.994	0.997	3.146	4.264	5.439
2	[92]	Unet	0.746	0.904	0.840	0.780	0.901	0.811	0.990	0.987	0.990	27.403	7.485	9.029
3	[104]	Ensemble	0.634	0.790	0.661	0.604	0.727	0.587	0.983	0.980	0.983	47.059	14.256	26.504

## Data Availability

Data available in publicly accessible repositories.
